# Supplementary figures and images for: Carboxylic acids that drive mosquito attraction to humans activate ionotropic receptors
Source: PLoS Negl Trop Dis. 2023 Jun 20;17(6):e0011402. doi: 10.1371/journal.pntd.0011402 (PMC10313001; doi:10.1371/journal.pntd.0011402)

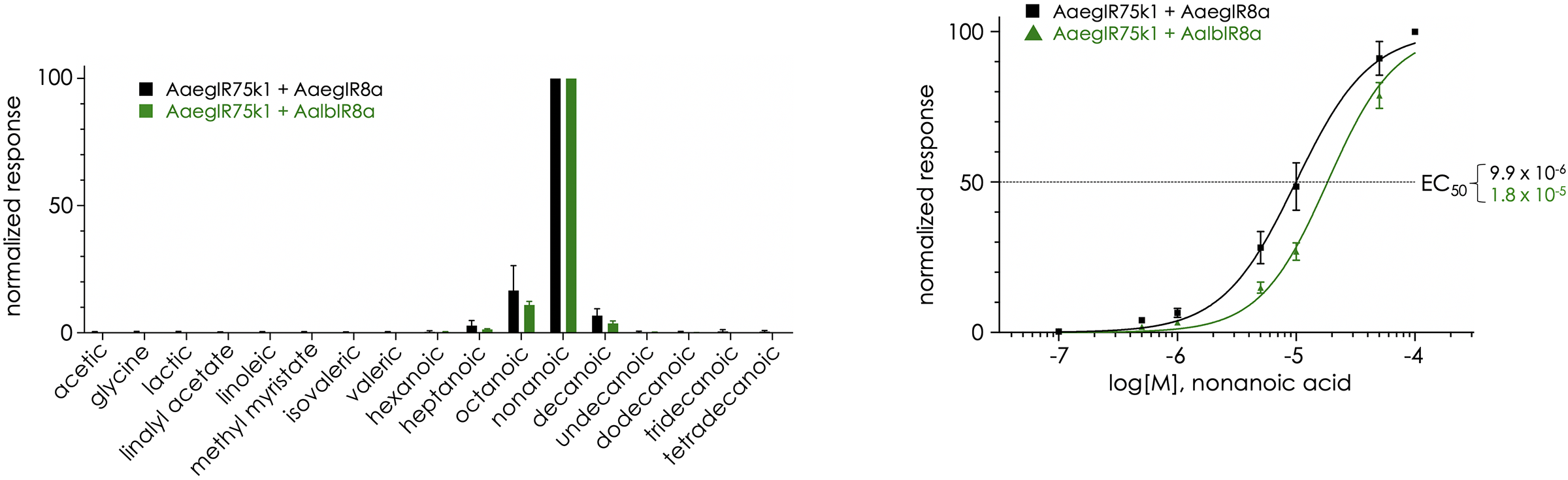

Supplement: S1 Fig — Left: AaegIR75k1 in combination with coreceptors AaegIR8a (black) or AalbIR8a (green) responds with highest efficacy to nonanoic acid. Right: AaegIR75k responded with greater sensitivity to nonanoic acid in the conspecific configuration. Raw data is provided in S3 Table. N = 10 oocytes per blend or compound at each concentration. (TIF) [file pntd.0011402.s001.tif]

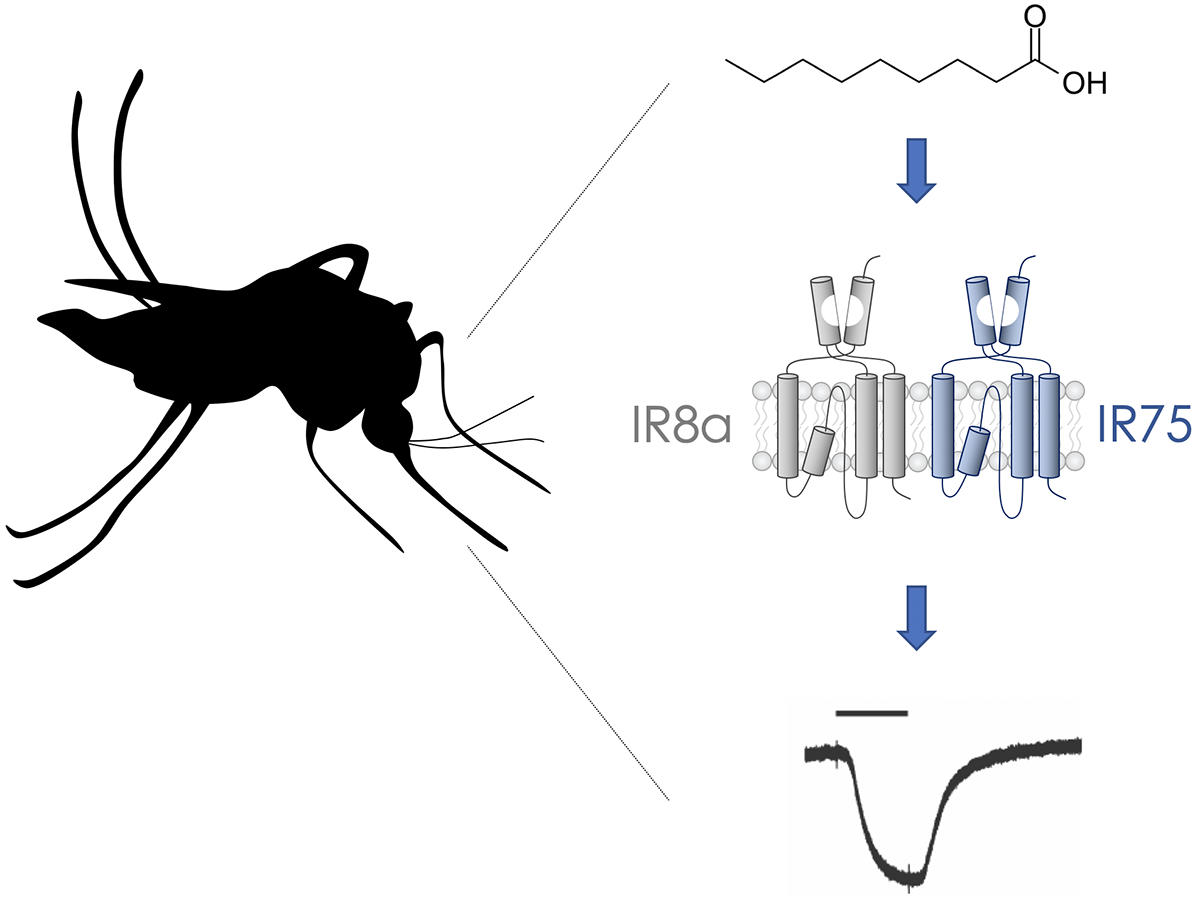

Supplement: S2 Fig — (TIF) [file pntd.0011402.s002.tif]
